# Melatonin supplementation in multiple sclerosis: a systematic review of clinical, imaging, biomarker, and safety outcomes

**DOI:** 10.3389/fneur.2026.1889519

**Published:** 2026-09-25

**Authors:** Yehia Nabil, Alina Ghazou, Sara Abodabesh, Anas Mansour, Ahmed Adel Mohamed, Salma Allam, Nourhan Eid, Yara Ibrahim Wahba, Ragda Mamoun MohamedAhmed Mamoun, Aya Samy, Kerolos E. Hanna, Ahmed Noufal, Enas Kamal Abdalaziz Seyed, Mostafa Meshref

**Affiliations:** 1 Faculty of Medicine, Zagazig University, Zagazig, Egypt; 2 Internship, Princess Basma Hospital, Ministry of Health, Irbid, Jordan; 3 Faculty of Medicine, Mansoura University, Mansoura, Egypt; 4 Faculty of Medicine, Al-Azhar University, Cairo, Egypt; 5 Faculty of Medicine, Galala University, Suez, Egypt; 6 Faculty of Medicine, Misr University for Science and Technology (MUST), Giza, Egypt; 7 Faculty of Pharmacy, Ahram Canadian University, Giza, Egypt; 8 Faculty of Medicine, Al-Neelain University, Khartoum, Sudan; 9 Department of Neurology, Faculty of Medicine, Al-Azhar University, Cairo, Egypt

**Keywords:** clinical outcomes, inflammation, melatonin, multiple sclerosis, oxidative stress, systematic review

## Abstract

**Purpose:**

Melatonin may act on immune, oxidative, and circadian pathways relevant to multiple sclerosis (MS), yet its clinical benefits and safety profile remain unclear. This systematic review assessed the efficacy and safety of oral melatonin supplementation in adults with MS.

**Methods:**

We searched MEDLINE (via PubMed), Scopus, Web of Science Core Collection, and the Cochrane Central Register of Controlled Trials (CENTRAL) from inception through August 15, 2026, and included randomized and prospective non-randomized intervention studies. Two reviewers independently selected studies, extracted data, and assessed risk of bias using RoB 2 or ROBINS-I. Given substantial clinical and methodological heterogeneity, we synthesized findings without meta-analysis, following SWiM guidance, and rated certainty of evidence with GRADE. We registered the protocol in PROSPERO (CRD420251167112).

**Results:**

We included 10 independent studies, reported across 17 publications, covering 435 participants. Nine were randomized trials (two of them crossover designs), and one was a prospective non-randomized intervention study. Melatonin doses spanned 0.5–300 mg/day, with treatment durations from 2 weeks to 14 months. The evidence did not show reductions in relapse activity, disability progression, or new MRI activity. Small studies suggested possible improvements in sleep, fatigue, pain, physical function, cognition, mood, autonomic function, body composition, and inflammatory, metabolic, or oxidative-stress biomarkers, but these results were inconsistent and rarely replicated. Doses of 0.5–25 mg/day were generally well tolerated, though some participants discontinued treatment and adverse-event reporting remained incomplete. At 300 mg/day, three of four melatonin-treated participants developed hypertransaminasemia, which resolved after withdrawal and recurred on rechallenge in one case. Certainty of evidence was low or very low for every outcome assessed.

**Conclusion:**

Current evidence does not support a disease-modifying effect of melatonin in MS. Any symptomatic or biomarker benefits need confirmation in larger, well-designed trials, and high-dose melatonin may carry meaningful hepatic risk.

## 1 Introduction

Multiple sclerosis (MS) is a chronic immune-mediated disease of the central nervous system marked by inflammatory demyelination, neuroaxonal injury, and neurodegeneration. Genetic, environmental, and immunological factors interact in producing disease susceptibility. Clinical expression varies widely: some patients follow a relapsing course, others a progressive one, and symptoms span motor, sensory, visual, cognitive, and autonomic domains, along with fatigue. Focal inflammatory activity and diffuse neurodegeneration both drive the accumulation of neurological disability (1–3).

The pineal gland synthesizes melatonin, an endogenous indoleamine, under circadian control. Beyond regulating sleep–wake rhythms, melatonin carries antioxidant, anti-inflammatory, immunomodulatory, and potentially neuroprotective properties (4). The “seasonal paradox” of MS disease activity supports its biological relevance to the disease. At temperate latitudes, vitamin D concentrations generally peak in spring and summer due to increased ultraviolet exposure, and given vitamin D’s immunoregulatory effects, relapse activity would be expected to drop during those months. Several cohorts instead report the opposite: more relapses in spring and summer, fewer in autumn and winter, though this pattern does not hold consistently across geographical settings. Melatonin secretion rises with longer nights and typically peaks in autumn and winter, tracking the relapse pattern these cohorts describe more closely than vitamin D does. A longitudinal cohort of relapsing–remitting MS patients found circulating melatonin levels inversely associated with clinical disease activity, offering epidemiological support for a link between photoperiod-dependent melatonin signaling and MS relapse biology (5, 6). These observational findings establish biological plausibility, not proof that exogenous melatonin prevents relapses.

Melatonin’s immunological rationale in MS goes beyond nonspecific suppression of individual inflammatory biomarkers. MS pathogenesis involves an imbalance between pathogenic CD4 + T-cell subsets, particularly Th1 and Th17 cells, and regulatory populations, including Foxp3 + regulatory T cells and IL-10-producing type 1 regulatory T cells. Pro-inflammatory cytokines like IL-1β, IL-6, and TNF-α build an inflammatory environment that activates and propagates effector T cells, while IL-10 counter-regulates. Melatonin signaling through the MT1 receptor, encoded by MTNR1A, can inhibit Th17 differentiation by modulating the REV-ERBα–NFIL3–RORγt pathway. MT1-dependent ERK1/2 signaling and ROR-α activation simultaneously promote the differentiation and function of IL-10-producing regulatory T cells (5, 6). Melatonin, then, potentially shifts the effector-to-regulatory T-cell balance rather than simply reducing a handful of unrelated circulating cytokines.

Experimental autoimmune encephalomyelitis (EAE) models back this immunoregulatory mechanism with preclinical evidence. Melatonin administration ameliorates EAE disease during both induction and established phases, reduces peripheral and central Th1 and Th17 responses, and increases Foxp3 + regulatory T-cell frequency and IL-10 production in the central nervous system. It also reduces the effector-memory T-cell population, shifts CD44 expression toward a more regulatory phenotype, and cuts inflammatory-cell infiltration into the central nervous system (7). Together, these findings point to a potential role for melatonin in limiting the initiation and spread of autoimmune attacks relevant to inflammatory disease activity and relapse biology.

The cuprizone model offers a distinct, non-redundant line of preclinical evidence. Unlike EAE, cuprizone induces oligodendrocyte injury and demyelination largely independent of peripheral autoimmune activation, which lets researchers study intrinsic myelin injury and repair directly. Melatonin reduces demyelination, supports remyelination, and attenuates oxidative and mitochondrial injury during both the demyelination and recovery phases of this model (8). Other experiments show reduced cuprizone-associated microgliosis and astrogliosis, along with greater preservation or recovery of mature oligodendrocytes in the corpus callosum, after melatonin administration (9). The EAE and cuprizone models together suggest complementary mechanisms: melatonin dampens autoimmune inflammation in EAE and protects oligodendrocytes while supporting myelin repair in the cuprizone model. These findings remain preclinical, though, and cannot be assumed to predict clinical efficacy in people with MS.

Human evidence lags well behind the mechanistic literature, in both maturity and consistency. Clinical trials have tested melatonin doses from roughly 0.5–25 mg/day over periods spanning 2 weeks to 12 months, tracking outcomes as varied as oxidative stress, inflammatory cytokines, relapse activity, sleep, fatigue, mood, cognitive function, physical performance, autonomic function, body composition, and neuroimaging measures (10–21). Some small trials found reductions in TNF-α, IL-1β, IL-6, and oxidative-stress markers; others found improvements in selected sleep, fatigue, cognitive, balance, gait, muscle-strength, or body-composition outcomes. Replication remains inconsistent. Many studies lacked power or tracked multiple outcomes without a clearly prioritized clinical endpoint. The four publications by Jallouli and colleagues draw on the same 12-week four-arm randomized trial; they represent one trial, not four (13, 17–19). Parkinson’s disease sleep research has tested melatonin across several randomized controlled trials. MS research sits at an earlier stage of clinical development despite its strong mechanistic rationale (22).

Safety conclusions have to account for the wide range of doses studied. Most low-to-moderate-dose MS studies reported acceptable short-term tolerability. The phase I/II MELATOMS-1 randomized trial tells a different story: it tested adjunctive melatonin at 300 mg/day in eight participants with primary progressive MS on stable ocrelizumab therapy, and researchers stopped it early after hypertransaminasemia developed in three of four melatonin-treated participants, versus none of four on placebo. Liver abnormalities resolved once participants stopped melatonin, and the abnormality recurred after rechallenge in one participant, strengthening the case for a causal relationship (23). These findings cannot be generalized beyond this small, highly selected, polymedicated population, but they rule out calling melatonin uniformly safe across its full dose range and underline the need to distinguish conventional supplementation from high-dose pharmacological administration.

A previous systematic review flagged potential symptomatic and biomarker benefits but found a small, heterogeneous clinical evidence base (10). Since then, additional clinical reports and new safety evidence have emerged, yet uncertainty persists around the independence of overlapping publications, the optimal dose and treatment duration, and the clinical relevance of reported outcomes. This systematic review updates the synthesis of human interventional evidence on melatonin supplementation in MS, covering clinical, functional, sleep-related, neuropsychological, imaging, biomarker, autonomic, and safety outcomes. It also characterizes heterogeneity in study design, dose, duration, and outcome selection, and identifies priorities for future clinical research.

## 2 Methods

This systematic review followed the Preferred Reporting Items for Systematic Reviews and Meta-Analyses (PRISMA) 2020 guidelines (24). The protocol was prospectively registered in the International Prospective Register of Systematic Reviews (PROSPERO) under registration number CRD420251167112.

### 2.1 Information sources and search strategy

We searched MEDLINE via PubMed, Scopus, Web of Science Core Collection, and the Cochrane Central Register of Controlled Trials (CENTRAL) from database inception to August 15, 2026. We also checked the reference lists of eligible articles and relevant reviews and tracked forward citations to identify additional reports. The search strategy combined controlled vocabulary, including Medical Subject Headings, with free-text terms relating to “multiple sclerosis,” “melatonin,” and relevant synonyms, adapted to each database’s indexing requirements. We imposed no restrictions on publication date or language. Supplementary Table S1 provides the complete reproducible search strategies and the number of records retrieved from each database.

### 2.2 Selection criteria

Eligible studies included adults aged 18 years or older with a confirmed MS diagnosis (relapsing–remitting, secondary progressive, primary progressive, or other definite relapsing or progressive forms) per the diagnostic criteria in use at the time. Mixed neurological populations qualified only when MS-participant data were reported separately. We excluded studies limited to clinically isolated syndrome without a confirmed MS diagnosis.

The intervention of interest was exogenous oral melatonin, alone or as an adjunct to stable disease-modifying or symptomatic therapy, in immediate-release or prolonged-release form, with no minimum treatment duration. We classified doses as low (≤3 mg/day), intermediate (>3–25 mg/day), or high (>25 mg/day). We excluded melatonin receptor agonists, multicomponent interventions where melatonin’s independent effect could not be isolated, and studies measuring endogenous melatonin without administering it.

Eligible comparators included placebo, usual care or no adjunctive melatonin, a different melatonin dose, or participants’ pre-intervention measurements in single-arm studies, in either parallel-group or crossover designs. For crossover trials, we extracted treatment sequence, period effects, washout, and carryover when reported.

We included randomized controlled trials (parallel-group and crossover) and prospective non-randomized intervention studies. We excluded retrospective observational studies, case–control studies without melatonin administration, cross-sectional studies of endogenous melatonin, case reports, case series, reviews, editorials, letters lacking primary data, animal studies, *in vitro* studies, and conference abstracts without sufficient methodological and outcome data.

### 2.3 Outcomes of interest included

Studies had to report at least one outcome relevant to melatonin’s efficacy or safety, across prespecified domains: clinical disease activity and disability (relapses, annualized relapse rate, EDSS, other disability measures); patient-reported and symptomatic outcomes (sleep, fatigue, pain, nocturia, mood, depression, anxiety, cognition, quality of life); physical and functional outcomes (gait, balance, fall risk, strength, dexterity, mobility); neuroimaging (T2 and gadolinium-enhancing lesions, lesion burden, atrophy, other MRI measures); inflammatory and immunological biomarkers (TNF-α, IL-1β, IL-6, IL-10, other cytokines or immune-cell markers); oxidative-stress and metabolic biomarkers (malondialdehyde, lipid hydroperoxides, total oxidant and antioxidant status, superoxide dismutase, glutathione, nitric oxide metabolites); autonomic, circadian, and pharmacokinetic outcomes (heart-rate variability, serum or urinary melatonin); and safety outcomes (adverse events, discontinuation, laboratory abnormalities, liver-enzyme elevations, suspected drug interactions). A study stayed eligible without every domain, as long as it covered at least one.

### 2.4 Study selection and data extraction

We imported all retrieved records into EndNote X9 (Clarivate Analytics, Philadelphia, PA, United States) and removed duplicates through a combination of automated and manual checks, then uploaded the deduplicated records to Rayyan for screening (25). Two reviewers (A. S and R. M. M. M) independently screened titles and abstracts. Any report either reviewer flagged as potentially eligible went to independent full-text assessment by the same two reviewers, who documented reasons for exclusion at that stage. We resolved disagreements through discussion and, when we could not reach consensus, consulted a third reviewer (Y. N). A PRISMA 2020 flow diagram documents the selection process.

Two reviewers (N. E and Y. I. W) independently extracted data using a standardized, pilot-tested Microsoft Excel form. Study-level information included author, publication year, country, clinical setting, study design, registration number, recruitment period, funding source, sample size, MS phenotype, diagnostic criteria, participant age and sex, disease duration, baseline disability, disease-modifying therapy, and concomitant medications.

Intervention data included melatonin formulation, dose, administration schedule, timing relative to sleep, treatment duration, adherence, and co-interventions. We also extracted comparator characteristics and, for crossover studies, treatment sequence and washout information.

For each outcome, the reviewers extracted the measurement instrument, assessment time point, number of participants analyzed, summary data, between-group effect estimate, corresponding 95% confidence interval, and reported adverse events. Safety extraction additionally covered the nature, severity, timing, reversibility, and suspected causality of adverse events; treatment withdrawal or rechallenge; hepatic laboratory results; and potentially interacting concomitant medications.

### 2.5 Identification of multiple reports from the same study

We treated the underlying study or trial, not the individual publication, as the unit of inclusion. We considered reports to originate from the same study when they shared a trial-registration number or showed substantial agreement in recruitment setting, recruitment period, participant characteristics, sample size, intervention dose, treatment duration, and investigator group.

We linked multiple publications from the same study as companion reports, drawing core study characteristics from the most complete report and supplementing outcome data from the associated publications. We counted participants and study characteristics only once, regardless of how many reports covered them. This mattered particularly for the multiple publications by Jallouli and colleagues and the potentially overlapping reports by Yosefifard and colleagues and Adamczyk-Sowa and colleagues.

### 2.6 Risk-of-bias assessment

Two reviewers (Y. I. W and A. S) independently assessed risk of bias, resolving disagreements by consensus or by consulting a third reviewer.

We evaluated randomized parallel-group trials using the Cochrane Risk of Bias 2 tool across the domains of bias arising from the randomization process, deviations from intended interventions, missing outcome data, outcome measurement, and selection of the reported result (26). We evaluated crossover trials using the RoB 2 crossover-trial variant, including period and carryover effects. Risk-of-bias judgments were outcome-specific, categorized as low risk of bias, some concerns, or high risk of bias.

We assessed prospective non-randomized intervention studies using the Risk Of Bias In Non-randomized Studies–of Interventions tool (ROBINS-I) (27), evaluating confounding, participant selection, intervention classification, deviations from intended interventions, missing data, outcome measurement, and selection of the reported result. Overall judgments fell into low, moderate, serious, or critical risk of bias, or insufficient information.

Where several publications came from the same underlying study, we assessed risk of bias at the study and outcome level rather than separately for each publication.

### 2.7 Data synthesis and statistical analysis

Substantial clinical and methodological heterogeneity, combined with the limited number of independent studies contributing to each outcome, made statistical pooling inappropriate. We instead synthesized the evidence using a structured synthesis without meta-analysis, following the SWiM reporting guideline (28).

We grouped studies by study design, melatonin dose category, treatment duration, MS phenotype, and outcome domain. For each outcome, the synthesis prioritized between-group effect estimates with 95% confidence intervals; where those were not available, we summarized post-intervention group values, changes from baseline, or within-participant estimates from crossover studies as reported.

We categorized the direction of effect descriptively as favoring melatonin, showing no clear difference, or favoring the comparator, basing interpretation on the magnitude, precision, consistency, and risk of bias of the evidence rather than statistical significance alone. Results appear in structured tables and, where appropriate, non-pooled forest plots or effect-direction displays without an overall summary estimate.

Multiple reports from the same study contributed only once to each outcome domain. We examined differences in dose, duration, comparator, participant characteristics, outcome definition, measurement instrument, and assessment time point as potential sources of heterogeneity. We performed no meta-analysis, meta-regression, formal subgroup pooling, I^2^ calculation, or assessment of small-study effects, since the assumptions those analyses require were not met.

### 2.8 Certainty of evidence

We evaluated the certainty of evidence for clinically important outcome domains using the Grading of Recommendations Assessment, Development and Evaluation approach (29). Evidence from randomized trials started at high certainty; evidence from non-randomized intervention studies started at low certainty. We downgraded certainty for risk of bias, inconsistency, indirectness, imprecision, or publication bias, and upgraded it where applicable according to established GRADE criteria.

Certainty judgments counted independent studies rather than publications, so multiple reports from the same trial could not artificially inflate the apparent evidence base. We categorized the final certainty of evidence as high, moderate, low, or very low.

## 3 Results

### 3.1 Study selection and characteristics

The searches identified 1,044 records from four databases. After removal of 52 duplicate records, 992 records underwent title and abstract screening, of which 961 were excluded. Thirty-one reports were sought for retrieval; all were retrieved and assessed for eligibility. Fourteen reports were excluded because they described ongoing trials without available results (*n* = 5), were published only as a letter or conference abstract (*n* = 1), were review articles (*n* = 1), or evaluated an irrelevant intervention or population (*n* = 7). Seventeen reports representing 10 independent studies met the eligibility criteria and were included in the review (Figure 1).

**Figure 1:**
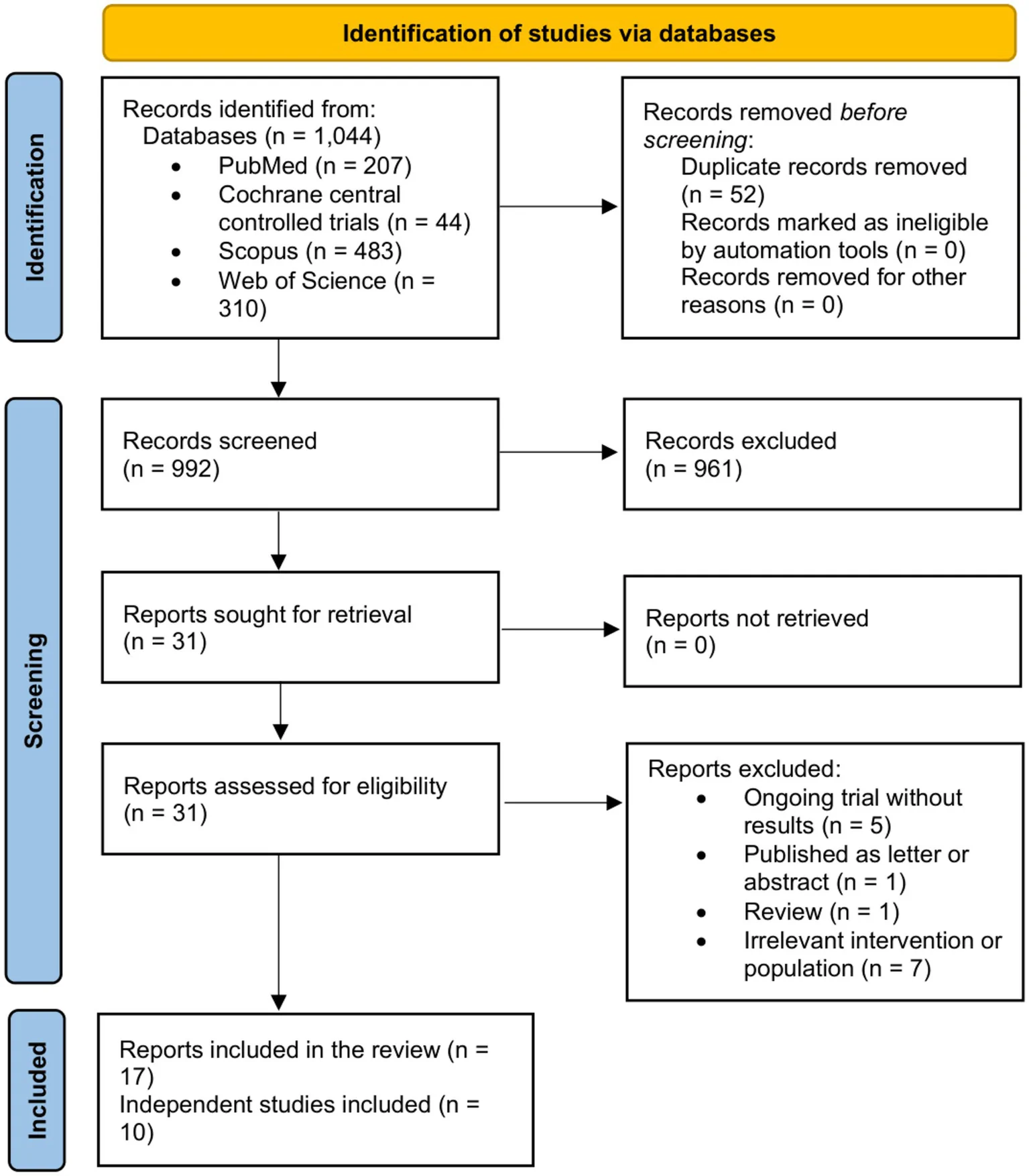
PRISMA flow diagram.

After companion reports were linked, four publications by Jallouli et al. were attributed to one 12-week four-arm randomized trial (PACTR202007465309582) in which 60 participants were randomized and 49 were analyzed. The three earlier reports presented outcomes from the melatonin-only and placebo arms, whereas the 2026 companion report presented the full trial, including training plus melatonin and training plus placebo arms (13, 17–19). Two publications by Yosefifard et al. reported different biomarkers from the same 50-participant trial (14, 30), and four publications by Adamczyk-Sowa et al. were treated as reports from a single prospective intervention cohort of 102 participants with MS (31–34). The 62-participant Iranian trial by Fuladi Targhi et al. had a distinct registration number, intervention duration, and outcome and was therefore treated as an independent study (12). Participants from these studies were counted once.

The 10 independent studies enrolled or randomized 435 participants with MS and were published between 2014 and 2026. Nine were randomized trials, including two crossover trials; one was a prospective non-randomized intervention study. Researchers conducted these studies in Poland, Iran, Mexico, the United Kingdom, the United States, Tunisia, and Spain (11–21, 23, 30–34).

Sample sizes ranged from eight participants in MELATOMS-1 to 102 in the Adamczyk-Sowa cohort. Most studies enrolled participants with relapsing–remitting or other relapsing forms of MS. Drake et al. included relapsing–remitting, secondary progressive, and primary progressive phenotypes; MELATOMS-1 enrolled only participants with primary progressive MS receiving stable ocrelizumab therapy (21, 23).

Melatonin doses ranged from 0.5 to 300 mg/day. Hsu et al. started treatment at 0.5 mg/night and escalated to 3 mg/night when required (16); Drake et al. evaluated 2 mg/night of prolonged-release melatonin (21). The Jallouli, Fuladi Targhi, Yosefifard, Roostaei, Smoot, and Adamczyk-Sowa studies used doses of 3–5 mg/day (12, 14, 15, 17–21, 30–34). Sánchez-López et al. administered 25 mg/day (11); MELATOMS-1 evaluated 300 mg/day (23). Treatment duration ranged from 2 weeks per crossover period to 12 months; exposure during the high-dose phase of MELATOMS-1 ranged from 2 to 14 months.

Most randomized trials used placebo comparators. Smoot et al. compared 3 and 5 mg/day without a placebo arm; the Adamczyk-Sowa cohort used pre-intervention measurements and healthy reference participants instead. Outcomes spanned disease activity, disability, magnetic resonance imaging, sleep, nocturia, fatigue, pain, quality of life, cognition, physical function, autonomic function, biomarkers, melatonin exposure, and safety. Tables 1, 2 present study-level and baseline characteristics.

**Table 1:** Characteristics of the independent studies and linked reports.

Independent study/linked reports	Country; design	Participants	Population	Intervention	Comparator	Duration	Outcome domains	Main findings
Adamczyk-Sowa cohort *Linked reports: 2014a, 2014b, 2016a, 2016b*	Poland; prospective non-randomized pre-post intervention study	102 participants with MS; healthy reference participants varied by report (15–20)	RRMS and progressive MS; DMT-defined subgroups	Melatonin 5 mg/day added to existing management	Pre-intervention values; healthy participants were external references, not a concurrent untreated MS comparator	90 days	Oxidative/metabolic biomarkers; sleep; fatigue; quality of life	Several oxidative markers and selected sleep or psychological measures improved in some subgroups. Causal interpretation is limited by the uncontrolled design, subgroup multiplicity, and overlapping reports.
Roostaei et al. (15)	Iran; double-blind parallel-group placebo-controlled RCT	26 randomized (13/13); 25 completed	RRMS	Melatonin 3 mg/night	Placebo	12 months	Relapses; EDSS; MSFC; fatigue; depression; MRI	No clear between-group benefit for relapses, disability, global MSFC, or new T2/gadolinium-enhancing lesions. An exploratory cognitive-fatigue subscore favored melatonin.
Fuladi Targhi et al. (12)	Iran; double-blind randomized placebo-controlled trial	62 randomized and analyzed (47/15)	RRMS receiving interferon beta	Melatonin 3 mg/night, 1 h before sleep	Placebo	8 weeks	Fatigue Severity Scale	Fatigue severity was lower after melatonin than placebo after baseline adjustment (*p* = 0.001). Interpretation is limited by markedly unequal allocation and incomplete reporting of allocation concealment, blinding implementation, prespecified analysis and adverse events.
Yosefifard trial *Linked reports: 2019 and 2020*	Iran; randomized placebo-controlled trial	50 randomized (25/25)	RRMS receiving routine interferon treatment	Melatonin 3 mg/day	Placebo	24 weeks	IL-1beta; TNF-alpha; serum interferon-beta1; vitamin B12	IL-1beta decreased versus placebo, whereas TNF-alpha showed no clear difference. A companion report described increases in serum interferon-beta1 and vitamin B12.
Sánchez-López et al. (11)	Mexico; double-blind parallel-group placebo-controlled RCT	36 randomized (18/18); 33 completed (17/16)	RRMS receiving interferon beta-1b	Melatonin 25 mg/day	Placebo	6 months	Inflammatory and oxidative biomarkers; EDSS; relapse; depression; safety	TNF-alpha, IL-1beta, IL-6 and selected oxidative-stress markers decreased, without clear improvement in EDSS or depression. Sleepiness was reported by approximately 23% of melatonin recipients.
Drake et al. (21) (MeNiMS)	United Kingdom; double-blind placebo-controlled crossover RCT	31 randomized; 26 completed	RRMS, SPMS and PPMS with nocturia	Prolonged-release melatonin 2 mg/night	Placebo; participants served as their own controls	2 weeks per period with a 4-week washout	Nocturia; lower urinary tract symptoms; sleep; quality of life; EDSS; safety	Melatonin did not clearly reduce nocturnal voids or improve urinary symptoms, sleep, quality of life, or EDSS.
Hsu et al. (16)	United States; randomized placebo-controlled crossover pilot trial	30 completed; valid paired actigraphy in 24 and patient-reported outcomes in 27	26 RRMS, 2 SPMS and 2 PPMS with sleep disturbance	Melatonin 0.5 mg/night, increased to 3 mg from day 4 if needed	Placebo; participants served as their own controls	2 weeks per period; no washout	Actigraphy; insomnia; sleep quality; daytime sleepiness; fatigue; walking; mood	Mean total sleep time was 7.0 versus 6.7 h (approximately 18 min; *p* = 0.03). Sleep efficiency and patient-reported outcomes showed no clear differences. No side effects were reported.
Smoot et al. (20)	United States; randomized dose-blinded exploratory parallel-group trial	30 randomized and analyzed (15/15); 23 completed	Relapsing MS receiving oral DMT	Melatonin 3 mg/day	Melatonin 5 mg/day; no placebo or untreated arm	12 months	Serum/urinary melatonin; fatigue; sleep; quality of life; disability; relapse; safety	The 5-mg dose produced higher average serum and urinary melatonin exposure, but no clear between-dose symptomatic benefit. Five participants discontinued melatonin because of adverse events.
Jallouli trial *Linked reports: 2024 (Behav Brain Res), 2025 (Metab Brain Dis), 2025 (J Diet Suppl), 2026 (Brain Res Bull)*	Tunisia; four-arm double-blind parallel-group placebo-controlled RCT	60 randomized (15 per arm); 49 analyzed (11 training + melatonin/11 training + placebo/15 melatonin/12 placebo)	RRMS	Melatonin 3 mg/night, with or without self-paced concurrent training	Placebo, with or without the same training program	12 weeks	Gait; balance; strength; dexterity; cognition; mood; sleep; fatigue; pain; HRV; oxidative stress; inflammation; lipid profile; body composition	Functional, sleep-related, autonomic and oxidative outcomes favored melatonin in earlier reports. The 2026 companion report found additional antioxidant, LDL-C, leukocyte and body-composition effects in the training comparison. Interpretation is limited by attrition, per-protocol analysis, extensive outcome testing, a training co-intervention, and reporting across four companion publications.
Bejarano et al. (23) (MELATOMS-1)	Spain; multicenter phase I/II double-blind placebo-controlled RCT	8 randomized (4/4) before interruption	PPMS receiving stable ocrelizumab	Melatonin 300 mg/day	Placebo	Intended follow-up 2 years; observed follow-up ranged from 2 to 14 months	Hepatic safety; adverse events; drug–drug interaction assessment	Hypertransaminasemia occurred in 3/4 melatonin-treated participants and 0/4 placebo-treated participants, resolved after withdrawal, and recurred after rechallenge in one participant. The high-dose phase was stopped and unblinded.

The unit of inclusion is the underlying study, not the publication. Jallouli, Yosefifard, and Adamczyk-Sowa companion reports are therefore grouped and participants are counted once. DMT, disease-modifying therapy; EDSS, Expanded Disability Status Scale; HRV, heart-rate variability; MRI, magnetic resonance imaging; MSFC, Multiple Sclerosis Functional Composite; PPMS, primary progressive MS; RCT, randomized controlled trial; RRMS, relapsing–remitting MS; SPMS, secondary progressive MS.

**Table 2:** Baseline characteristics of participants, organized by independent study.

Study	Analysis group	*N*	Age, years (reported statistic)	Female	BMI (reported statistic)	MS phenotype	Disease duration, years	Baseline EDSS	DMT/background therapy
Adamczyk-Sowa cohort	All MS participants across reported DMT subgroups	102	Subgroup means 33.5–54.2	69/102 (67.6%)	–	85 RRMS; 17 SPMS/PPMS	Subgroup means 1.85–20.88	Subgroup means 1.85–5.68	81 receiving DMT; 21 pretreated/not yet receiving DMT
Roostaei et al. (15)	Melatonin	13	33.3 ± 7.6 (mean ± SD)	9/13 (69.2%)	–	RRMS	6.0 ± 4.7	1.8 ± 1.5	Yes
	Placebo	13 randomized; 12 completed	34.5 ± 8.2 (mean ± SD)	12/12 analyzed (100%)	–	RRMS	2.8 ± 1.7	0.77 ± 1.0	Yes
Fuladi Targhi et al. (12)	Melatonin	47	37.87 ± 8.96 (mean ± SD)	40/47 (85.1%)	–	RRMS	–	Eligibility required EDSS 0–5.5	Interferon beta
	Placebo	15	38.86 ± 8.74 (mean ± SD)	11/15 (73.3%)	–	RRMS	–	Eligibility required EDSS 0–5.5	Interferon beta
Yosefifard trial	Melatonin	25	37.76 ± 6.45 (reported as mean ± SE)	22/25 (88%)	–	RRMS	–	Eligibility required EDSS 0–5	Routine interferon treatment
	Placebo	25	37.96 ± 6.88 (reported as mean ± SE)	23/25 (92%)	–	RRMS	–	Eligibility required EDSS 0–5	Routine interferon treatment
Sánchez-López et al. (11)	Melatonin	18 randomized; 17 completed	39 ± 6.5 (mean ± SD)	71%	23.8 ± 3.2	RRMS	7.8 ± 3.9	1.9 ± 1.0	Interferon beta-1b
	Placebo	18 randomized; 16 completed	40 ± 5.5 (mean ± SD)	75%	24.1 ± 3.3	RRMS	8.1 ± 4.6	2.1 ± 1.2	Interferon beta-1b
Drake et al. (21)	Crossover cohort	31 randomized; 26 completed	54.8 (range 34–69)	18/31 (58.1%)	–	11 RRMS; 15 SPMS; 5 PPMS	–	Mean 4.2; median 4.0 (range 1.5–8.0)	5/11 participants with RRMS were receiving DMT
Hsu et al. (16)	Crossover cohort	30 completed	46.7 ± 1.8 (mean ± SEM)	26/30 (86.7%)	28.8 ± 1.4 (mean ± SEM)	26 RRMS; 2 SPMS; 2 PPMS	9.7 ± 1.0 (mean ± SEM)	Median 2.0 (IQR 2.5; range 0–6.5)	25/30 receiving DMT; 5 none
Smoot et al. (20)	Melatonin 3 mg	15	54 ± 8 (mean ± SD)	12/15 (80%)	–	Relapsing MS	–	–	All receiving oral DMT
	Melatonin 5 mg	15	52 ± 9 (mean ± SD)	11/15 (73%)	–	Relapsing MS	–	–	All receiving oral DMT
Jallouli trial	Melatonin (analyzed cohort)	15	34.67 ± 10.93 (mean ± SD)	11/15 (73.3%)	23.96 ± 4.29	RRMS	Median 6.0 (IQR 7.0)	Median 1.0 (IQR 1.5)	8 first-line; 7 s-line
	Placebo (analyzed cohort)	12	36.83 ± 8.07 (mean ± SD)	8/12 (66.7%)	22.96 ± 4.39	RRMS	Median 4.5 (IQR 5.0)	Median 2.75 (IQR 1.5)	9 first-line; 3 s-line
	Training + melatonin (analyzed cohort)	11	28.36 ± 8.66 (mean ± SD)	6/11 (54.5%)	–	RRMS	6.59 ± 5.16	1.73 ± 1.31	55% first-line; 45% second-line
	Training + placebo (analyzed cohort)	11	33.09 ± 10.26 (mean ± SD)	6/11 (54.5%)	–	RRMS	6.82 ± 5.16	1.96 ± 1.25	82% first-line; 18% second-line
Bejarano et al. (23)	Melatonin 300 mg	4	38–57 (individual ages)	3/4 (75%)	–	PPMS	5–17	5.5–7.0	Stable ocrelizumab; multiple concomitant medications
	Placebo	4	48–59 (individual ages)	1/4 (25%)	–	PPMS	5–16	5.0–6.5	Stable ocrelizumab; concomitant medications

Values are reproduced using the statistic reported by each study; SEM, SD, IQR and range are therefore explicitly identified and should not be treated as interchangeable. For crossover trials, participants served as their own controls and are shown once. Jallouli baseline values are shown for the 49 analyzed participants across the four trial arms; 60 participants were randomized. The Adamczyk-Sowa values are summarized once across overlapping companion reports; healthy reference participants were not included as eligible MS intervention participants. BMI, body mass index; DMT, disease-modifying therapy; EDSS, Expanded Disability Status Scale; IQR, interquartile range; PPMS, primary progressive MS; RRMS, relapsing–remitting MS; SD, standard deviation; SEM, standard error of the mean; SPMS, secondary progressive MS.

### 3.2 Risk of bias of included studies

Figures 2, 3 show the RoB 2 and ROBINS-I assessments. Eight randomized trials raised some concerns overall; the Smoot trial carried high overall risk of bias. No randomized trial reached low overall risk. The main concerns centered on incomplete reporting of randomization or allocation concealment, missing outcome data, and limited detail on prespecified analyses and outcome selection.

**Figure 2:**
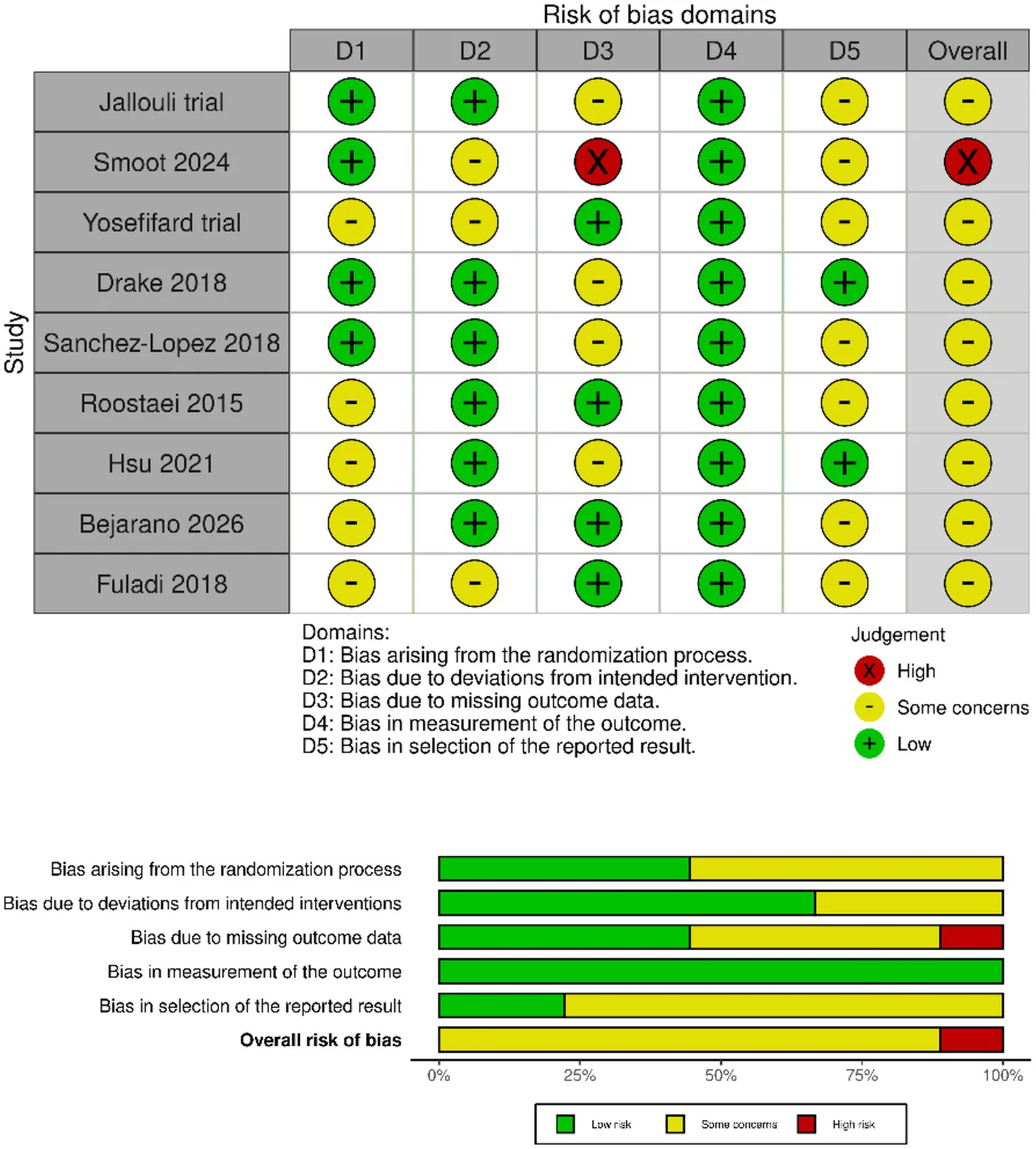
RoB 2 assessment of the randomized trials.

**Figure 3:**
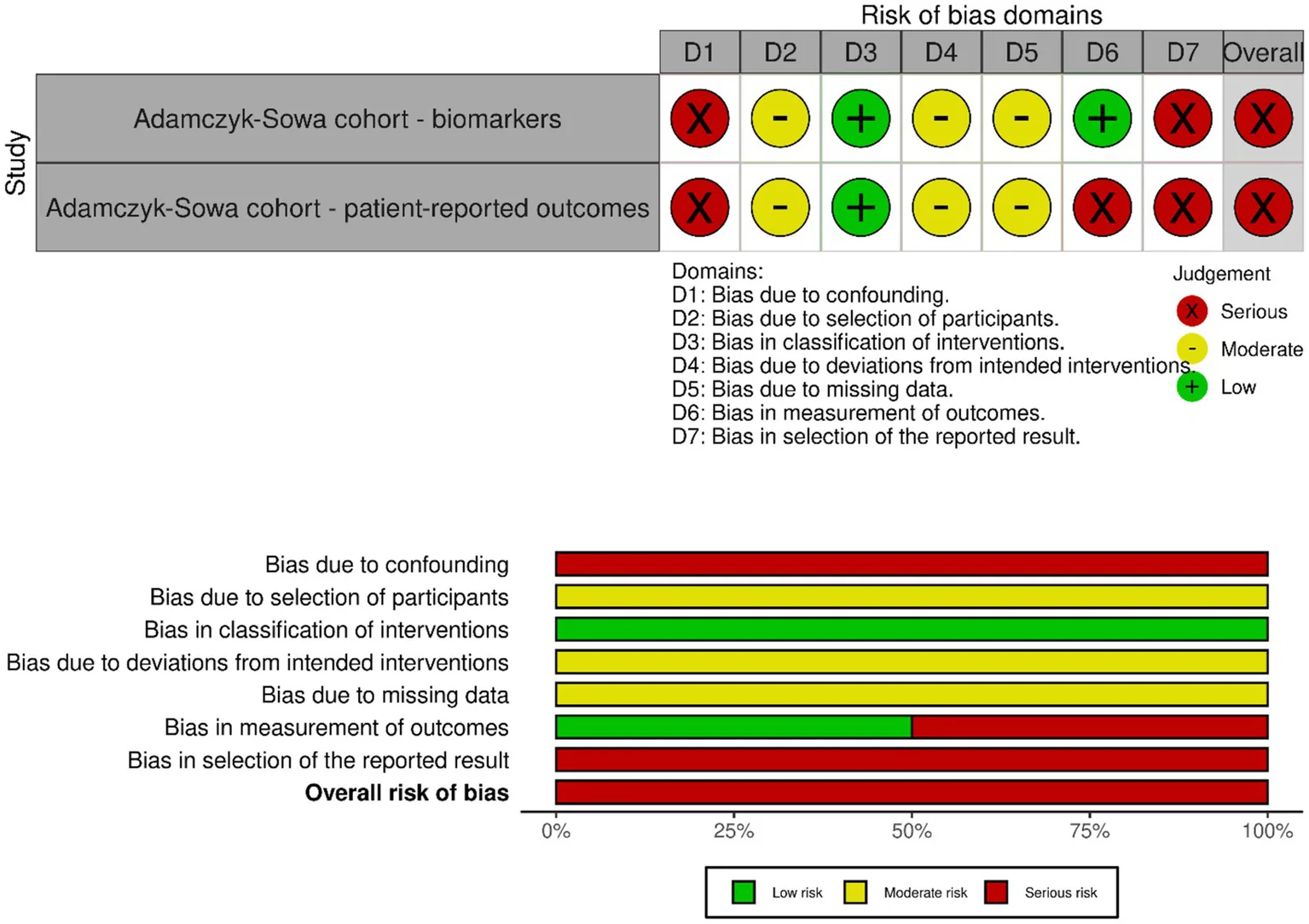
ROBINS-I assessment of the non-randomized intervention study.

For the crossover trials: Hsu et al. skipped a washout period, leaving uncertainty about period and carryover effects, though melatonin’s short half-life may reduce pharmacological carryover (16). Drake et al. built in a four-week washout and assessed period and carryover effects, but attrition remained a concern (21). The Jallouli trial used computer-generated block randomization and sealed opaque envelopes, but 11 of 60 randomized participants were excluded from the per-protocol analysis, no intention-to-treat analysis was reported, and numerous outcomes appeared across four companion publications (13, 17–19). Fuladi Targhi et al. described a randomized double-blind trial, but reporting of allocation concealment, blinding implementation, and prespecified analyses was limited, and the 47:15 group imbalance reduced confidence in the randomization process (12). Treatment discontinuation drove high risk of bias from missing outcome data in the Smoot trial (20). Limited reporting of allocation concealment or prespecified analyses affected the Yosefifard and Roostaei trials (14, 15, 30).

The Adamczyk-Sowa study carried serious overall risk of bias for both biomarker and patient-reported outcomes. All participants with MS received melatonin, no concurrent untreated MS group existed, and the analyses did not adequately control for confounding by disease-modifying therapy. The healthy reference participants did not offer an appropriate counterfactual for estimating melatonin’s effect (31–34).

### 3.3 Disease activity, disability, and neuroimaging

In the 12-month Roostaei trial, 3 mg/night of melatonin showed no association with between-group differences in relapse number, Expanded Disability Status Scale score, global Multiple Sclerosis Functional Composite score, new T2-lesion number or volume, or gadolinium-enhancing lesion number or volume (15). An exploratory analysis favored melatonin for the cognitive component of the Modified Fatigue Impact Scale, but not for overall fatigue or the principal clinical and imaging outcomes.

Sánchez-López et al. found no between-group improvement in disability or other reported clinical efficacy measures despite changes in inflammatory and oxidative biomarkers (11). No relapses occurred in the Smoot trial, but the absence of a placebo or untreated comparator ruled out attributing this to melatonin (20). Researchers interrupted MELATOMS-1 before they could meaningfully evaluate efficacy, disability-progression, or imaging outcomes (23).

Taken together, the available evidence did not demonstrate a reduction in relapse activity, disability progression, or new magnetic resonance imaging activity.

### 3.4 Sleep, nocturia, fatigue, pain, and quality of life

In the Hsu crossover trial, actigraphy-measured mean total sleep time reached 7.0 h during melatonin treatment and 6.7 h during placebo treatment (*p* = 0.03). The roughly 18-min difference fell below the prespecified 20-min threshold for clinical significance. Sleep efficiency reached 84.7% with melatonin and 83.2% with placebo (*p* = 0.06). Insomnia severity, subjective sleep quality, daytime sleepiness, fatigue, mood, and walking-related patient-reported outcomes did not differ between treatments (16).

The Jallouli trial reported improvements in Pittsburgh Sleep Quality Index scores, sleep duration and efficiency, fatigue, and neuropathic pain after 12 weeks of 3 mg/night melatonin (17, 18). These results came from a single trial and remain unreplicated.

Fuladi Targhi et al. randomized 62 Iranian participants with relapsing–remitting MS on interferon beta therapy: 47 to 3 mg/night of melatonin, 15 to placebo, for 8 weeks. After adjusting for baseline values, the melatonin group scored lower on the Fatigue Severity Scale than placebo (30.42 ± 12.40 versus 41.00 ± 12.66; *p* = 0.001) (12). The markedly unequal allocation and limited reporting of trial methods warrant cautious interpretation.

In MeNiMS, 2 mg/night of prolonged-release melatonin did not reduce nocturnal voiding or improve lower urinary tract symptoms, sleep quality, or nocturia-related or MS-related quality of life among the 26 participants who completed the crossover trial (21).

The Adamczyk-Sowa cohort reported improvements in Athens Insomnia Scale scores in selected disease-modifying-therapy subgroups, but not consistently across all subgroups. The physical component of the Multiple Sclerosis Impact Scale did not improve consistently, though selected psychological scores improved in some interferon-treated subgroups (31, 32). In the Smoot trial, neither 3 nor 5 mg/day improved fatigue, sleep quality, patient-determined disability, or MS-related quality of life, and no between-dose differences emerged (20).

### 3.5 Physical, cognitive, and mood outcomes

Across the Jallouli companion reports, melatonin correlated with greater walking speed and stride length, lower center-of-pressure velocity during dynamic balance assessment, greater knee-flexor and knee-extensor peak torque, improved static balance, and shorter Nine-Hole Peg Test completion time (17, 19). The reports also described improvements in Montreal Cognitive Assessment, Symbol Digit Modalities Test, Trail Making Test, and total Hospital Anxiety and Depression Scale scores (19). These findings came from 27 analyzed participants across multiple outcomes.

Roostaei et al. found no between-group differences in the global Multiple Sclerosis Functional Composite, overall fatigue, or depression (15). Hsu et al. likewise found no differences in actigraphy-measured step count or self-reported walking ability (16).

### 3.6 Inflammatory and immunological biomarkers

Sánchez-López et al. reported reductions in serum TNF-α, IL-1β, and IL-6 after 6 months of 25 mg/day melatonin compared with placebo (11). In the Yosefifard trial, 3 mg/day for 24 weeks reduced IL-1β but did not significantly affect TNF-α (14). A companion report from the same trial described increases in serum interferon-β1 and vitamin B12 concentrations (30).

Melatonin affected selected inflammatory biomarkers, but the findings varied across analytes and came without consistent clinical or imaging benefits.

### 3.7 Oxidative-stress and metabolic biomarkers

The Adamczyk-Sowa reports described reductions in total oxidant status, malondialdehyde, and lipid hydroperoxides, and increases in selected antioxidant measures, in some disease-modifying-therapy subgroups. Homocysteine did not change consistently, and ceruloplasmin findings varied between subgroups (31–34). These analyses rested primarily on uncontrolled pre-post comparisons.

Sánchez-López et al. reported reductions in malondialdehyde and other oxidative-stress markers compared with placebo (11). The Jallouli trial reported reductions in malondialdehyde and advanced oxidation protein products and an increase in reduced glutathione (18). Differences in laboratory methods, biomarker definitions, doses, and comparators ruled out a consistent quantitative synthesis.

The 2026 Jallouli companion report evaluated the full four-arm trial. Among 49 analyzed participants, training plus melatonin, compared with the same training plus placebo, increased reduced glutathione, total bilirubin, and uric acid; reduced malondialdehyde, advanced oxidation protein products, LDL cholesterol, and selected leukocyte counts; increased lean mass; and reduced fat mass and body mass index (13). No clear between-group differences were detected for HDL cholesterol, total cholesterol, triglycerides, glucose, insulin, cortisol, renal-function markers, cellular-damage markers, or C-reactive protein. Because this report arose from the same Jallouli trial and included a training co-intervention, it adds outcome domains but not independent replication.

### 3.8 Autonomic and pharmacokinetic outcomes

In one Jallouli companion report, melatonin increased the root mean square of successive differences and the high-frequency component of heart-rate variability, and reduced the low-frequency component, compared with placebo (18).

In the Smoot trial, 5 mg/day produced higher average serum melatonin and urinary 6-sulfatoxymelatonin concentrations than 3 mg/day. Serum and urinary concentrations correlated, but this difference in exposure did not translate into between-dose differences in fatigue, sleep, quality of life, or patient-determined disability (20).

### 3.9 Safety

No consistent serious toxicity pattern emerged at conventional doses of 0.5–25 mg/day, though adverse-event reporting varied across studies. Hsu et al. reported no treatment-related adverse events (16), and neither the Drake nor Roostaei trials showed a clear safety signal (15, 21). Sánchez-López et al. reported sleepiness in roughly 23% of melatonin-treated participants but no serious adverse events (11). The Jallouli reports stated that no adverse events occurred (17–19); the 2026 companion report skipped systematic adverse-event results, though post-randomization withdrawals traced mainly to training attendance, COVID-19-related illness, or personal commitments (13). Fuladi Targhi et al. left detailed adverse-event results unreported (12).

In the Smoot trial, five participants discontinued melatonin because of adverse events: two on 3 mg/day and three on 5 mg/day. One participant in the 5-mg group developed severe focal spongiotic dermatitis; the remaining events leading to discontinuation were mild or moderate. Researchers considered one serious cardiac event unrelated to melatonin (20).

MELATOMS-1 identified a distinct hepatic safety signal at 300 mg/day. Hypertransaminasemia occurred in three of four melatonin-treated participants and none of four placebo-treated participants, prompting researchers to interrupt and unblind the high-dose phase. Liver-enzyme abnormalities improved after melatonin withdrawal, and one participant experienced recurrent elevations following reintroduction (23). All three affected participants were polymedicated women taking medications that shared hepatic metabolic pathways with melatonin. The small sample and *post hoc* nature of the interaction analysis ruled out reliable identification of sex- or medication-specific risk factors.

### 3.10 Certainty of evidence

Table 3 summarizes the GRADE judgments. Certainty was very low for disease activity, disability and imaging; sleep and related symptoms; fatigue, physical function, cognition and mood; inflammatory and oxidative biomarkers; and hepatic safety at 300 mg/day. Certainty regarding safety at conventional doses was low.

**Table 3:** GRADE certainty of evidence by clinically important outcome domain.

Outcome domain	No. of studies (independent evidence base)	Certainty assessment				Certainty of the evidence
		Risk of bias	Inconsistency	Indirectness	Imprecision	
Relapse activity, disability and MRI	Principally Roostaei 2015 (1 small RCT; 26 randomized, 25 completed); supportive clinical observations from other trials were not designed to establish disease modification	Serious: small trial, baseline imbalance and limited reporting	Not assessable/serious: too few directly comparable studies	Not serious: for the directly studied RRMS population; applicability to progressive MS is limited	Very serious: few events and extremely small sample	⊕◯◯◯ Very low
Sleep and related symptoms	Hsu 2021, Jallouli trial, Drake 2018, Smoot 2024 and Adamczyk-Sowa cohort; heterogeneous designs, doses and instruments	Serious: attrition, carryover uncertainty, uncontrolled data and selective/multiple outcome reporting	Serious: objective and subjective findings were inconsistent	Serious: different sleep disorders, instruments and treatment comparisons	Serious: small studies and imprecise estimates	⊕◯◯◯ Very low
Fatigue, physical function, cognition and mood	Jallouli trial, Fuladi Targhi 2018, Roostaei 2015, Hsu 2021, Smoot 2024 and Adamczyk-Sowa cohort	Serious: extensive multiplicity, small samples and inclusion of uncontrolled analyses	Serious: favorable and null results across studies	Serious: diverse constructs, instruments, time points and doses	Very serious: most outcomes rely on one small trial without replication	⊕◯◯◯ Very low
Inflammatory and oxidative biomarkers	Sanchez-Lopez 2018, Yosefifard trial, Jallouli trial and Adamczyk-Sowa cohort	Serious: multiple comparisons and one uncontrolled cohort	Serious: effects differed across biomarkers and studies	Serious: surrogate outcomes with uncertain linkage to clinical benefit	Serious: small samples and no robust replication for individual markers	⊕◯◯◯ Very low
Safety at conventional doses (0.5–25 mg/day)	Nine independent studies; 427 enrolled/randomized participants in total, with variable exposure and adverse-event reporting	Serious: adverse-event ascertainment and reporting were inconsistent	Not serious: for absence of a repeated serious toxicity pattern, but discontinuations occurred	Serious: short follow-up in several studies and selected trial populations	Serious: samples were inadequate to exclude uncommon harms	⊕ ⊕ **◯◯** Low
Hepatic safety at 300 mg/day	Bejarano 2026 only: 8 randomized; 4 exposed to high-dose melatonin	Serious: early stopping and *post hoc* interaction analysis, although randomized comparison and dechallenge/rechallenge support the signal	Not assessable: single trial	Serious: PPMS participants on ocrelizumab with polypharmacy	Very serious: only four exposed participants	⊕◯◯◯ Very low

MRI, magnetic resonance imaging; PPMS, primary progressive multiple sclerosis; RCT, randomized controlled trial; RRMS, relapsing–remitting multiple sclerosis.

Downgrading reflected risk of bias, inconsistency, indirectness, and imprecision. Most clinical outcome domains relied on one or a few small trials, used heterogeneous instruments, and lacked independent replication. The 2026 Jallouli report expanded the outcome set from an existing trial rather than supplying independent confirmation. Biomarker evidence was limited by multiple comparisons, methodological heterogeneity, and uncertain clinical relevance. Conventional-dose safety evidence was constrained by small samples, short follow-up, and inconsistent adverse-event reporting. Dechallenge and rechallenge observations supported the high-dose hepatic finding, but it rested on only four exposed participants.

The evidence overall did not establish a disease-modifying effect of melatonin in MS. Potential symptomatic, functional, autonomic, and biomarker effects remain uncertain given the limited and heterogeneous evidence base.

## 4 Discussion

This systematic review identified 17 reports representing 10 independent intervention studies and 435 participants with MS. Small samples, wide variation in melatonin dose and treatment duration, heterogeneous outcome selection, and multiple publications from the same participant cohorts characterized the evidence base. Three findings stand out. The available evidence did not demonstrate that melatonin reduces relapse activity, disability progression, or magnetic resonance imaging activity. Selected studies reported improvements in sleep, fatigue, physical function, autonomic measures, body composition, and inflammatory, metabolic, or oxidative biomarkers, but these findings were inconsistent and generally lacked independent replication. Safety appeared dose-dependent: conventional doses showed no consistent pattern of serious toxicity, while 300 mg/day produced a clinically important hepatic signal in MELATOMS-1. Certainty was low for conventional-dose safety and very low across every other evaluated domain.

This review extends the previous systematic review by Morsali et al., which identified potential symptomatic and biomarker benefits but flagged the small and heterogeneous clinical literature (10). Our synthesis incorporates subsequently published fatigue, functional, metabolic, body-composition, pharmacokinetic, and high-dose safety data, distinguishes reports from independent studies, and evaluates the evidence using design-appropriate risk-of-bias tools and GRADE. Linking companion publications materially changed the interpretation: the four Jallouli reports represent one trial rather than four independent confirmations, the two Yosefifard publications report outcomes from one participant sample, and the four Adamczyk-Sowa publications appear to describe a single prospective cohort. Fuladi Targhi et al. contributed one distinct Iranian randomized trial (12). Counting publications without accounting for these relationships would overstate both the size and reproducibility of the evidence base.

The mechanistic rationale for melatonin in MS outpaces the clinical evidence. Observational work has linked higher endogenous melatonin concentrations with lower seasonal disease activity, and experimental studies have demonstrated effects on pathogenic and regulatory T-cell populations, oxidative stress, oligodendrocyte injury, and remyelination (5–9). These findings do not establish that exogenous supplementation modifies disease activity in humans. Endogenous circadian melatonin exposure, pharmacological supplementation, and the immune environment of experimental models represent biologically distinct contexts.

Roostaei et al. gave the most direct evaluation of disease activity and found no between-group benefit for relapses, disability, global functional performance, or magnetic resonance imaging outcomes after 12 months (15). Other studies either were not designed to test disease modification or were too small to yield informative estimates. The absence of relapses in the Smoot study cannot be attributed to melatonin: both groups received active supplementation and the trial included no untreated or placebo comparator (20). MELATOMS-1 was interrupted before efficacy could be meaningfully evaluated (23). The current evidence neither demonstrates a disease-modifying effect nor excludes a small effect that these underpowered studies simply could not detect.

Sleep findings came out inconsistent. Hsu et al. identified a roughly 18-min increase in actigraphy-measured total sleep time, but this difference fell below the study’s prespecified threshold for clinical significance, and it came without significant improvements in subjective sleep, fatigue, mood, or daytime sleepiness (16). Drake et al. found no benefit for nocturia, sleep quality, or related quality-of-life outcomes (21); Smoot et al. observed no symptomatic difference between 3- and 5-mg doses (20). The improvements in insomnia scores reported in the Adamczyk-Sowa cohort stayed confined to selected treatment subgroups, and we cannot reliably separate them from temporal changes, regression to the mean, or confounding by disease-modifying therapy (31, 32).

The broad functional and neuropsychological benefits across the Jallouli publications all arose from one trial testing numerous gait, balance, strength, dexterity, cognitive, mood, sleep, pain, autonomic, biochemical, metabolic, and body-composition outcomes (13, 17–19). Fuladi Targhi et al. reported a reduction in fatigue after 8 weeks, but the unequal allocation and limited reporting restrict confidence in that finding (12). Even when individual comparisons reach statistical significance, extensive outcome testing raises the probability of chance findings unless researchers address multiplicity prospectively. The absence of comparable benefits in the Roostaei, Hsu, and Smoot studies further limits confidence in the reproducibility of these effects (15, 16, 20). These results work better as hypothesis-generating than as evidence supporting routine symptomatic treatment.

Heterogeneity in the intended therapeutic indication offers another explanation for the inconsistency across studies. Researchers evaluated melatonin as a treatment for sleep disturbance, nocturia, fatigue and related symptoms, as a biomarker-modifying adjunct, and as a potential disease-modifying therapy. These questions involve different target populations, mechanisms, and clinically relevant endpoints, and we should not treat them as estimates of a single underlying treatment effect. A modest objective sleep benefit among participants selected for sleep disturbance, for instance, is not necessarily inconsistent with the absence of benefit in a trial targeting nocturia or in an unselected MS population (16, 21). Future trials should define one primary therapeutic indication and preferentially enroll participants with the corresponding baseline impairment; including participants without the target symptom risks diluting a clinically meaningful treatment effect.

Several studies reported reductions in pro-inflammatory cytokines or oxidative-stress markers (11, 13, 14, 18, 30–34). These findings align directionally with the antioxidant and immunomodulatory mechanisms described in experimental models. Effects still were not consistent across individual biomarkers, and laboratory methods, doses, background therapies, and study designs differed substantially. The uncontrolled design and multiple subgroup comparisons in the Adamczyk-Sowa cohort further restrict causal interpretation. The 2026 Jallouli companion report also combined melatonin with concurrent training, so its findings should not be treated as independent confirmation of melatonin-only effects.

Changes in circulating biomarkers did not consistently come with improvements in relapses, disability, imaging, or patient-reported outcomes. Serum cytokines and oxidative-stress measures remain surrogate outcomes, and their relationship to compartmentalized central nervous system inflammation and neurodegeneration stays uncertain. We should not interpret biomarker improvement as evidence of clinical efficacy. Future mechanistic studies should prespecify a limited number of biomarkers and test whether changes correlate with clinically meaningful outcomes, rather than reporting isolated laboratory differences.

The safety findings demand a clear distinction between conventional supplementation and high-dose pharmacological administration. Across studies evaluating 0.5–25 mg/day, no consistent pattern of serious treatment-related toxicity emerged, though adverse-event ascertainment stayed incomplete and sample sizes were too small to exclude uncommon harms. Sleepiness occurred in roughly 23% of melatonin-treated participants in Sánchez-López et al., and five participants discontinued treatment in the Smoot trial, including one with severe focal spongiotic dermatitis (11, 20). Fuladi Targhi et al. and the 2026 Jallouli companion report did not provide systematic adverse-event results (12, 13). We should describe conventional doses as reasonably tolerated in small studies, not uniformly safe.

MELATOMS-1 delivers a qualitatively different safety finding. Hypertransaminasemia occurred in three of four participants receiving 300 mg/day and in none of four receiving placebo; liver abnormalities improved after withdrawal and recurred after reintroduction in one participant (23). The randomized comparison, temporal relationship, dechallenge, and rechallenge strengthen causal concern. Still, the exposed sample was extremely small, and all affected participants were polymedicated women taking medications with hepatic pathways shared with melatonin. The incidence of toxicity and the independent contributions of dose, sex, ocrelizumab, and concomitant medications remain uncertain.

After the 300-mg phase was interrupted, researchers amended the MELATOMS-1 protocol to restart treatment at 100 mg/day, with prespecified dose de-escalation and intensified hepatic monitoring (23). This protocol adaptation should not be read as evidence that the lower pharmacological doses are safe or effective: the amended phase remains ongoing and has not yet provided comparative efficacy data.

These findings challenge the assumption that the established tolerability of over-the-counter melatonin extrapolates to pharmacological doses. People with MS should not use high-dose melatonin outside a clinical trial. Future dose-escalation studies should incorporate systematic medication-interaction assessment, predefined hepatic stopping rules, and frequent liver-function monitoring.

Daily dose alone may not tell the whole story either. Melatonin carries both sleep-promoting and chronobiotic effects, and its biological response depends partly on the timing of administration relative to endogenous circadian phase. Human phase-response studies have shown that the direction and magnitude of circadian shifts vary according to both administration time and dose (35). The included MS studies did not consistently characterize chronotype, dim-light melatonin onset, evening light exposure, or administration time relative to habitual sleep, so participants receiving the same nominal dose may have received melatonin at different biological times. The finding that 5 mg/day produced higher serum and urinary melatonin exposure than 3 mg/day without superior symptomatic outcomes further suggests that greater systemic exposure does not necessarily translate into greater clinical benefit (20). Future studies should prespecify formulation and administration time, document habitual sleep and light exposure, and consider measuring circadian phase in a mechanistic subset.

Clinicians should not currently recommend melatonin as a disease-modifying treatment or as a strategy to prevent MS relapses. The evidence also falls short of supporting an MS-specific recommendation for fatigue, cognitive impairment, balance dysfunction, pain, or autonomic symptoms.

For patients with MS and coexisting insomnia considering conventional-dose melatonin, clinicians can note that objective sleep duration improved modestly in one small crossover trial, while subjective sleep and daytime outcomes did not consistently improve. Treatment decisions should stay individualized, following assessment of other contributors to sleep disturbance, including nocturia, pain, mood disorders, sleep-disordered breathing, restless legs syndrome, medication effects, and circadian disruption. Expectations should stay modest, and patients should discontinue treatment if it produces no clinically meaningful benefit.

The hepatic findings from MELATOMS-1 do not generalize directly to conventional doses, but they carry clinical relevance when considering high-dose preparations, compounded formulations, or patients receiving multiple hepatically metabolized medications. Dose deserves treatment as an essential component of any safety statement, not lumping all melatonin exposure into a single intervention.

This review’s strengths include an updated search of bibliographic databases, explicit separation of independent studies from companion publications, inclusion of randomized and prospective non-randomized intervention evidence, and design-specific assessment using RoB 2 and ROBINS-I. A structured synthesis without meta-analysis avoided producing a potentially misleading pooled estimate from clinically and methodologically incompatible outcomes. GRADE assessment also distinguished uncertainty regarding efficacy, conventional-dose tolerability, and high-dose hepatic safety.

The evidence base comprised only 10 independent studies, most small and incompletely reported. Considerable heterogeneity in population, MS phenotype, dose, formulation, treatment duration, comparator, background therapy, co-interventions, and outcome measurement ruled out quantitative pooling. Single studies reported many outcomes, and multiple comparisons were common. Inconsistent adverse-event definitions and ascertainment limit conclusions about both common and uncommon harms. We cannot exclude the possibility of unpublished or selectively reported findings, and the small number of studies prevented formal assessment of publication bias. Study-level data could not determine whether treatment effects or adverse events differed according to MS phenotype, disability, sex, circadian characteristics, disease-modifying therapy, or concomitant medications.

Formulation quality adds a further source of uncertainty. Several reports gave limited information regarding immediate- versus prolonged-release preparation, pharmaceutical quality, batch verification, or actual melatonin content. This matters because laboratory testing of commercially available melatonin products has demonstrated substantial variation between measured and labelled content, along with marked lot-to-lot variability (36). These findings do not generalize to every preparation used in the included trials, but they underscore that future studies should use pharmaceutical-grade melatonin, report release characteristics and excipients, and independently verify product content and stability. Readers should regard the dose categories used in this review as descriptive, not as validated pharmacological thresholds.

The evidence supports a staged clinical-development strategy rather than jumping straight to a large disease-modification trial. The top priority: an adequately powered, placebo-controlled trial targeting a clearly defined symptomatic indication (particularly objectively verified sleep disturbance), using a standardized formulation, biologically informed administration time, and one prespecified clinically meaningful primary outcome. Parallel dose-finding and pharmacokinetic studies should determine whether exposure correlates with symptom response, while systematically assessing treatment discontinuation, liver function, and concomitant medications. A larger trial evaluating relapse activity, disability progression, or magnetic resonance imaging outcomes would only be justified once earlier studies establish an acceptable dose, reproducible target engagement, and a credible clinical or imaging signal. Given the current very-low-certainty evidence, undertaking a definitive disease-modification trial without these intermediate steps would be premature.

Trials designed to evaluate disease modification would need substantially larger samples, longer follow-up, and prespecified relapse, disability, and magnetic resonance imaging endpoints. Changes in circulating cytokines or oxidative biomarkers alone should not justify such trials. Researchers can embed mechanistic substudies within clinical trials, but they should prespecify biomarkers and link them analytically to clinical or imaging outcomes.

Across both symptomatic and disease-modifying trials, priorities should include concealed randomization, robust blinding, strategies to minimize attrition, prospective registration of primary and secondary outcomes, multiplicity control, and publication of integrated trial reports. Safety monitoring should include systematic adverse-event collection, liver-function testing at pharmacological doses, documentation of concomitant medications, and predefined dose-modification and stopping criteria. These measures will determine whether the biological rationale for melatonin translates into clinically meaningful benefit without unacceptable harm.

## 5 Conclusion

Exogenous melatonin has not been shown to reduce relapse activity, disability progression, or MRI activity in multiple sclerosis. Individual studies reported potential benefits for sleep, fatigue, physical and cognitive function, autonomic measures, body composition, and inflammatory, metabolic, or oxidative biomarkers, but findings were inconsistent, mostly came from single small trials, and lacked replication. Biomarker changes should not count as evidence of clinical efficacy.

Conventional doses (0.5–25 mg/day) appeared reasonably tolerated, though small samples and short follow-up limit firm safety conclusions. Hepatic toxicity at 300 mg/day shows this tolerability does not extend to pharmacological doses; people with MS should not use high-dose melatonin outside monitored trials.

Routine use as a disease-modifying or MS-specific symptomatic treatment is not supported. Future trials should first target clearly defined sleep disorders with standardized formulations and systematic safety monitoring, reserving disease-modification trials for after an acceptable dose and reproducible signal are established.

## Data Availability

The original contributions presented in the study are included in the article/Supplementary material, further inquiries can be directed to the corresponding authors.

## References

[1] Woo MS Engler JB Friese MA. The neuropathobiology of multiple sclerosis. Nat Rev Neurosci. (2024) 25:493–513. doi: 10.1038/s41583-024-00823-z 38789516

[2] Reich DS Lucchinetti CF Calabresi PA. Multiple sclerosis. N Engl J Med. (2018) 378:169–80. doi: 10.1056/NEJMra1401483 PMC6942519 29320652

[3] Olsson T Barcellos LF Alfredsson L. Interactions between genetic, lifestyle and environmental risk factors for multiple sclerosis. Nat Rev Neurol. (2016) 13:25–36. doi: 10.1038/nrneurol.2016.187, 27934854

[4] Hardeland R Cardinali DP Srinivasan V Spence DW Brown GM Pandi-Perumal SR. Melatonin-a pleiotropic, orchestrating regulator molecule. Prog Neurobiol. (2011) 93:350–84. doi: 10.1016/j.pneurobio.2010.12.004 21193011

[5] Farez MF Mascanfroni ID Méndez-Huergo SP Yeste A Murugaiyan G Garo LP . Melatonin contributes to the seasonality of multiple sclerosis relapses. Cell. (2015) 162:1338–52. doi: 10.1016/j.cell.2015.08.025 PMC4570563 26359987

[6] Farez MF Calandri IL Correale J Quintana FJ. Anti-inflammatory effects of melatonin in multiple sclerosis. BioEssays. (2016) 38:1016–26. doi: 10.1002/bies.201600018 27561251

[7] Álvarez-Sánchez N Cruz-Chamorro I López-González A Utrilla JC Fernández-Santos JM Martínez-López A . Melatonin controls experimental autoimmune encephalomyelitis by altering the T effector/regulatory balance. Brain Behav Immun. (2015) 50:101–14. doi: 10.1016/j.bbi.2015.06.021 26130320

[8] Abo Taleb HA Alghamdi BS. Neuroprotective effects of melatonin during demyelination and Remyelination stages in a mouse model of multiple sclerosis. J Mol Neurosci. (2020) 70:386–402. doi: 10.1007/s12031-019-01425-6 31713152

[9] Alidadi M Omidi N Abdi M Mohammadi M Shabani M Kashani IR. Melatonin ameliorates astrogliosis and microgliosis in a cuprizone demyelinating mouse model. Biochem Biophys Rep. (2025) 41:101929. doi: 10.1016/j.bbrep.2025.101929 PMC11803163 39926210

[10] Morsali S Sabahi Z Kakaei J Hakimzadeh Z Hamidi S Gholipour-khalili E . Clinical efficacy and safety of melatonin supplementation in multiple sclerosis: a systematic review. Inflammopharmacology. (2023) 31:2213–20. doi: 10.1007/s10787-023-01271-4 37429996

[11] Sánchez-López AL Ortiz GG Pacheco-Moises FP Mireles-Ramírez MA Bitzer-Quintero OK Delgado-Lara DLC . Efficacy of melatonin on serum pro-inflammatory cytokines and oxidative stress markers in relapsing remitting multiple sclerosis. Arch Med Res. (2018) 49:391–8. doi: 10.1016/j.arcmed.2018.12.004, 30595364

[12] Fuladi Targhi F Faraji F Maleki Rad AA Ghassami K Talaei A. Study the effect of melatonin on fatigue in patients with multiple sclerosis. J Arak Univ Med Sci. (2018) 21:67–75.

[13] Jallouli S Jallouli D Kallel C Sakka S Damak M Jaafar B . Benefits of 12-week self-paced training combined with melatonin supplementation on oxidative stress, inflammation, hyperlipidemia, and body composition in multiple sclerosis patients: a randomized controlled trial. Brain Res Bull. (2026) 243:111968. doi: 10.1016/j.brainresbull.2026.111968 42217742

[14] Yosefifard M Vaezi G Malekirad AA Faraji F Hojati V. A randomized control trial study to determine the effect of melatonin on serum levels of IL-1β and TNF-α in patients with multiple sclerosis. Iran J Allergy Asthma Immunol. (2019) 18:649–54. doi: 10.18502/ijaai.v18i6.2177, 32245308

[15] Roostaei T Sahraian MA Hajeaghaee S Gholipour T Togha M Siroos B . Impact of melatonin on motor, cognitive and neuroimaging indices in patients with multiple sclerosis. Iran J Allergy Asthma Immunol. (2015) 14:589–95. 26725556

[16] Hsu WY Anderson A Rowles W Peters KE Li V Stone KL . Effects of melatonin on sleep disturbances in multiple sclerosis: a randomized, controlled pilot study. Mult Scler J Exp Transl Clin. (2021) 7:20552173211048756. doi: 10.1177/20552173211048756 PMC8573503 34777854

[17] Jallouli S Ghroubi S Damak M Sakka S Elleuch MH Mhiri C . 12-week melatonin supplementation improved dynamic postural stability and walking performance in persons living with multiple sclerosis: a randomized controlled trial. Behav Brain Res. (2024) 476:115191. doi: 10.1016/j.bbr.2024.115191, 39122092

[18] Jallouli S Jallouli D Damak M Sakka S Ghroubi S Mhiri C . 12-week melatonin intake attenuates cardiac autonomic dysfunction and oxidative stress in multiple sclerosis patients: a randomized controlled trial. Metab Brain Dis. (2024) 40:52. doi: 10.1007/s11011-024-01428-2, 39630381

[19] Jallouli S Ghroubi S Bouattour N Sakka S Damak M Mhiri C . Effects of melatonin supplementation on muscle strength, manual dexterity, and postural balance in patients living with multiple sclerosis - a randomized controlled trial. J Diet Suppl. (2025) 22:236–61. doi: 10.1080/19390211.2024.2449030, 39849681

[20] Smoot K Gervasi-Follmar T Marginean H Chen C Cohan S. Impact of oral melatonin supplementation on urine and serum melatonin concentrations and quality-of-life measures in persons with relapsing multiple sclerosis. Mult Scler Relat Disord. (2024) 90:105799. doi: 10.1016/j.msard.2024.105799, 39126937

[21] Drake MJ Canham L Cotterill N Delgado D Homewood J Inglis K . Results of a randomized, double blind, placebo controlled, crossover trial of melatonin for treatment of nocturia in adults with multiple sclerosis (MeNiMS). BMC Neurol. (2018) 18:1–8. doi: 10.1186/s12883-018-1114-4, 30081859 PMC6091206

[22] Ma H Yan J Sun W Jiang M Zhang Y. Melatonin treatment for sleep disorders in Parkinson’s disease: a meta-analysis and systematic review. Front Aging Neurosci. (2022) 14:784314. doi: 10.3389/fnagi.2022.784314 PMC8855052 35185525

[23] Bejarano I Jiménez-Jorge S Lobo-Acosta MÁ Álvarez-López AI Rosso-Fernández CM López-Ruiz R . Hepatic safety of adjunctive high-dose melatonin in participants receiving Ocrelizumab for primary progressive multiple sclerosis: liver toxicity findings from a phase I/II randomised clinical trial (MELATOMS-1). CNS Drugs. (2026) 40:579–96. doi: 10.1007/s40263-025-01261-w PMC12988967 41706381

[24] Page MJ McKenzie JE Bossuyt PM Boutron I Hoffmann TC Mulrow CD . The PRISMA 2020 statement: an updated guideline for reporting systematic reviews. BMJ. (2021) 372. doi: 10.1136/bmj.n71 PMC8005924 33782057

[25] Ouzzani M Hammady H Fedorowicz Z Elmagarmid A. Rayyan-a web and mobile app for systematic reviews. Syst Rev. (2016) 5:1–10. doi: 10.1186/s13643-016-0384-4 PMC5139140 27919275

[26] Sterne JAC Savović J Page MJ Elbers RG Blencowe NS Boutron I . RoB 2: a revised tool for assessing risk of bias in randomised trials. BMJ. (2019) 366. doi: 10.1136/bmj.l4898 31462531

[27] Sterne JA Hernán MA Reeves BC Savović J Berkman ND Viswanathan M . ROBINS-I: a tool for assessing risk of bias in non-randomised studies of interventions. BMJ. (2016) 355. doi: 10.1136/bmj.i4919 PMC5062054 27733354

[28] Campbell M McKenzie JE Sowden A Katikireddi SV Brennan SE Ellis S . Synthesis without meta-analysis (SWiM) in systematic reviews: reporting guideline. BMJ. (2020) 368. doi: 10.1136/bmj.l6890 PMC7190266 31948937

[29] Guyatt G Oxman AD Akl EA Kunz R Vist G Brozek J . GRADE guidelines: 1. Introduction—GRADE evidence profiles and summary of findings tables. J Clin Epidemiol. (2011) 64:383–94. doi: 10.1016/j.jclinepi.2010.04.026 21195583

[30] Yosefi-Fard M Vaezi G Maleki-Rad AA Faraji F Hojati V. Effect of melatonin on serum levels of inf-1ß and vitamin B12 in patients with multiple sclerosis: a randomized controlled trial. Iran J Toxicol. (2020) 14:19–24. doi: 10.32598/ijt.14.1.19

[31] Adamczyk-Sowa M Pierzchala K Sowa P Mucha S Sadowska-Bartosz I Adamczyk J . Melatonin acts as antioxidant and improves sleep in MS patients. Neurochem Res. (2014) 39:1585–93. doi: 10.1007/s11064-014-1347-6, 24974099 PMC4122810

[32] Adamczyk-Sowa M Pierzchala K Sowa P Polaniak R Kukla M Hartel M. Influence of melatonin supplementation on serum antioxidative properties and impact of the quality of life in multiple sclerosis patients. J Physiol Pharmacol. (2014) 65:543–50. 25179086

[33] Adamczyk-Sowa M Sowa P Adamczyk J Niedziela N Misiolek H Owczarek M . Effect of melatonin supplementation on plasma lipid hydroperoxides, homocysteine concentration and chronic fatigue syndrome in multiple sclerosis patients treated with interferons-beta and mitoxantrone. J Physiol Pharmacol. (2016) 67:235–42. 27226183

[34] Adamczyk-Sowa M Sowa P Mucha S Zostawa J Mazur B Owczarek M . Changes in serum ceruloplasmin levels based on immunomodulatory treatments and melatonin supplementation in multiple sclerosis patients. Med Sci Monit. (2016) 22:2484–91. doi: 10.12659/MSM.895702, 27420299 PMC4958372

[35] Burgess HJ Revell VL Molina TA Eastman CI. Human phase response curves to three days of daily melatonin: 0.5 mg versus 3.0 mg. J Clin Endocrinol Metab. (2010) 95:3325–31. doi: 10.1210/jc.2009-2590 PMC2928909 20410229

[36] Erland LAE Saxena PK. Melatonin natural health products and supplements: presence of serotonin and significant variability of melatonin content. J Clin Sleep Med. (2017) 13:275–81. doi: 10.5664/jcsm.6462, 27855744 PMC5263083

