# Supplementary material for: Melatonin supplementation in multiple sclerosis: a systematic review of clinical, imaging, biomarker, and safety outcomes
Source: Front Neurol. 2026 Sep 25;17:1889519. doi: 10.3389/fneur.2026.1889519 (PMC13649473; doi:10.3389/fneur.2026.1889519)
Supplement: Supplementary file 1 [file Table_1.docx]

Supplementary table 1: Detailed search strategy

| **Database** | **Search strategy** | **No. of results** |
| --- | --- | --- |
| **MEDLINE via PubMed** | (  "Multiple Sclerosis"[Mesh]  OR "multiple sclerosis"[Title/Abstract]  OR "multiple scleroses"[Title/Abstract]  OR "disseminated sclerosis"[Title/Abstract]  OR "sclerosis multiplex"[Title/Abstract]  )  AND  (  "Melatonin"[Mesh]  OR melatonin[Title/Abstract]  OR melatonine[Title/Abstract]  OR "N-acetyl-5-methoxytryptamine"[Title/Abstract]  OR "N-acetyl-5-methoxy tryptamine"[Title/Abstract]  OR "5-methoxy-N-acetyltryptamine"[Title/Abstract]  ) | 207 |
| **Cochrane Central Register of Controlled Trials (CENTRAL)** | ("multiple sclerosis" OR "multiple scleroses" OR "disseminated sclerosis" OR "sclerosis multiplex")  AND  (melatonin OR melatonine OR "N-acetyl-5-methoxytryptamine" OR "N-acetyl-5-methoxy tryptamine" OR "5-methoxy-N-acetyltryptamine") | 44 |
| **Scopus** | TITLE-ABS-KEY(  "multiple sclerosis"  OR "multiple scleroses"  OR "disseminated sclerosis"  OR "sclerosis multiplex"  )  AND  TITLE-ABS-KEY(  melatonin  OR melatonine  OR "N-acetyl-5-methoxytryptamine"  OR "N-acetyl-5-methoxy tryptamine"  OR "5-methoxy-N-acetyltryptamine"  ) | 483 |
| **Web of Science Core Collection** | TS=(  "multiple sclerosis"  OR "multiple scleroses"  OR "disseminated sclerosis"  OR "sclerosis multiplex"  )  AND  TS=(  melatonin  OR melatonine  OR "N-acetyl-5-methoxytryptamine"  OR "N-acetyl-5-methoxy tryptamine"  OR "5-methoxy-N-acetyltryptamine"  ) | 310 |
| **Total** | | 1,044 |
